# Intermittent in the West

**Published:** 1835

**Authors:** 


### X.—Intermittent Fever in the West.

Intermittent fever appears to be prevailing extensively in the west. Why does not this malady appear in town as well as country? In Cincinnati we seldom have a case unless the person had visited the country. We speak of the thickly settled portions of the city—for the suburbs, especially the western, in the direction of the low alluvial shores of Mill-creek, have been partially affected. We confess ourselves unable to assign any satisfactory reason, for the exemption of cities from this disease; for there are, certainly, within most of them, various sources of malaria; and malignant remittents occasionally occur, even as epidemics.

As far as we have heard, the intermittent fever of the present autumn is mild; but there are exceptions, some of which, through the politeness of the attending physician, Dr. Ridgely, have fallen under our own observation. As these cases were of a remarkable character, we shall insert a notice of them.

Burlington is a small village on the Ohio river, in our own state, nearly opposite the mouth of Great Sandy, which separates Virginia from Kentucky. A family by the name of Cox, resided one mile below the village, on the north bank of the Ohio river. The shore is high, and exempt both from alluvial accumulations and collections of water; but on the opposite side of the river, above the mouth of the Big Sandy, there are several large ponds. The people on both sides of the Ohio, including those of the village of Burlington, were generally affected with intermittent fever. Among the rest, Mr. Cox and every member of his family, amounting in all to eight persons, were taken down. He, himself, in the course of the disease was seized with convulsions and delirium, of which he died. One of the children, laboring under the fever, became affected with symptoms of epidemic cholera, and died. Another laboring under the same fever, experienced an attack with convulsions, like the father, which terminated in hemiplegia, from which, however, it has nearly recovered. All these events happened at the same place. Soon afterwards the remaining members of the family removed to Cincinnati, and fell under the care of Dr. Ridgley. One of the children, a boy four or five years old, when the Dr. first saw him, appeared to be coming out of the cold stage. He was able to sit up in the bed and converse rationally. But soon after the Dr. left the house, he said he was dying, and in fact expired —having complained of severe pain in his bowels, a symptom which existed in the paroxysm of the preceding day. Not long afterwards, a daughter, two or three years older, laboring under the same form of fever, was attacked with convulsions, accompanied with hemiplegia, and after several repetitions, throughout the intervals of which she remained senseless, she expired. Two other children and the mother are recovering. One of these children, according to the statement of the mother, had a paroxysm of the fever, when it was but three days old. Of the two that died in the city, Dr. Ridgley was permitted to examine the body of one only, the boy, but had not an opportunity of inspecting the brain or spinal marrow. The mucous membrane of the stomach and bowels was free from inflammatory lesions. The liver was unusually firm, and of a leaden color. The spleen was dark colored, engorged, and enlarged.

The whole family had been treated, before they came to Cincinnati, with the sulphate of quinine, and bloodletting, both general and local, had been omitted. The Dr. and myself are of opinion, that the whole, at first, required the lancet; and suppose that to its omission, and the early and empyrical administration of the sulphate of quinine, the sinister termination of most of them might be fairly attributed. One of the children who is recovering, has sluggish bowels and a tumified abdomen. The treatment of this case is by purgatives, alternated with the sulphate; by which purging we propose to divert from the brain, and prevent a lesion of that organ, while the sulphate exerts its specific action in arresting the paroxysms.

When intermitting fever is epidemic, it can scarcely be doubted, that many patients are lost by the premature and indiscriminate administration of quinine, a medicine which affects the brain much more than the Peruvian bark in substance.

